# Delayed acute pancreatitis induced by nilotinib in a patient with chronic myeloid leukemia attaining sustained complete molecular response

**DOI:** 10.1002/jha2.21

**Published:** 2020-05-31

**Authors:** Shifang Wang, Sai Prasad Desikan, Jay Jeffrey, Charles McClain, Raman Desikan

**Affiliations:** ^1^ White River Health System Batesville Arizona USA

**Keywords:** CML leukemia, CML MDR, therapy

## Abstract

Advent of tyrosine kinase inhibitors (TKI) have revolutionized therapy of chronic myeloid leukemia. Imatinib was the first agent utilized in the therapy of CML. Nilotinib, a second generation TKI, results in an increase in number of patients achieving major molecular response at an earlier time point. Asymptomatic elevations in pancreatic enzyme is common and acute pancreatitis within weeks to months from start of therapy has been observed. Delayed onset pancreatitis has not been reported. We report a case of delayed onset pancreatitis in a patient with sustained complete molecular response. On account of the deep response, we were able to avoid starting alternate tyrosine kinase inhibitors that could also result in pancreatitis as a class effect.

## 1 INTRODUCTION

Chronic myeloid leukemia (CML) is a myeloproliferative neoplasm characterized by Philadelphia chromosome translocation (9:22) (q34; q11) and driven by the resultant fusion product, (BCR‐ABL1) a tyrosine kinase. Introduction of imatinib, a selective tyrosine kinase inhibitor of BCR‐ABL1, resulted in dramatic improvement in response and survival [1, 2]. Nilotinib, a second‐generation tyrosine kinase inhibitor, is more potent and specific for BCR‐ABL. When employed as front‐line therapy, it provides an increased and rapid response [3, 4]. We would like to report our observation of delayed onset acute pancreatitis in a patient attaining complete molecular response (MR4.5) (BCR‐ABL1 ≤ 0.0032%).

## 2 CASE PRESENTATION

**FIGURE 1:**
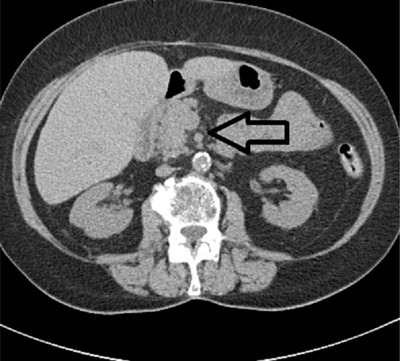
Black arrow indicates peripancreatic stranding consistent with pancreatitis

A 78‐year old Caucasian female was started on nilotinib as front‐line therapy in 2013. She tolerated nilotinib therapy, although asymptomatic elevations of lipase and amylase were noted to a peak of 354 U/L(23‐300 U/L) and 82 U/L(30‐110 U/L), respectively. Therapy was continued and elevations subsided without necessitating dose modification. Within 2 years of starting nilotinib, the patient achieved complete molecular remission of her CML. She continued nilotinib maintenance therapy for the next 2 years with regular surveillance showing continued response (MR 4.5). After 4 years of nilotinib therapy, she presented to the emergency department with a 7‐day history of abdominal pain in her right upper quadrant and epigastric area, which had acutely worsened over previous 3 to 4 days. On evaluation, she had tenderness to palpation in the epigastric region without guarding or rebound tenderness. Vitals at presentation showed a temperature of 97.4°F, heart rate of 80 bpm, blood pressure of 131/69, respiratory rate of 18/min, and oxygen saturation of 95% on room air. Laboratory evaluation was significant for elevated: amylase 154 U/L (30‐110 U/L; CTCAE Grade 1), lipase 1775 U/L (23‐300 U/L; CTCAE Grade 4), bilirubin 1.4 mg/dL (0.2‐1.3 mg/dL; CTCAE Grade1), AST 109 U/L (14‐36; CTCAE grade 1), ALT 194 (9‐52; CTCAE grade 2), and Alkaline phosphotase 245(38‐126; CTCAE grade 1). Serum triglycerides (149 mg/dL, 0‐149 mg/dL) and calcium (8.4 mg/dL) were normal at presentation. CT scan showed stranding of the pancreatic head, suggestive of pancreatitis. No changes were noted in the common bile duct, and the gallbladder was absent on account of a prior cholecystectomy (Figure 1). MRCP performed did not reveal abnormalities in biliary drainage including intrahepatic, extra hepatic, and common bile duct. The patient denied alcohol and recreational drug use, as well as abdominal trauma. A review of current medications with potential to cause acute pancreatitis was performed. The possible drugs implicated and classification based on Badalov et al were: glimepride (class IV), lisinopril (class Ia), furosemide (class Ia), metformin (class III), Aspirin (class III), Alendronate (class III), and nilotinib (class II) [15, 16].

Nilotinib was identified as the probable cause of drug induced pancreatitis and discontinued. The patient improved significantly with supportive care; on discharge after 4 days of hospitalization, the lipase level decreased to 353 U/L. On account of sustained complete molecular response to nilotinib (MR4.5), the patient was observed closely without any further therapy. Molecular remission has persisted for over 2 years on close monitoring.

## 3 DISCUSSION

Advent of tyrosine kinase inhibitor therapy in CML has improved response rates significantly. The majority of patients attain complete cytogenetic response (83%) and hence, more sensitive measurements are necessary to evaluate minimal residual disease. This has been accomplished by molecular testing with standardized real‐time quantitative polymerase chain reaction (RQ‐PCR) for detection of BCR‐ABL mRNA, wherein sensitivity is high for low‐level residual disease (10^−4^ to 10^−5^). Molecular response is assessed in accordance with the International Scale (IS) as the ratio of BCR‐ABL1 transcripts to ABL1 transcripts or other internationally recognized control transcripts, and reported as BCR‐ABL1 percent on a log scale [7]. The degree of molecular response correlates well with risk of progression. Nilotinib, when compared with imatinib, resulted in earlier and higher rates of major molecular response and lower risk of progression to accelerated phase or blast crisis. Long‐term follow up confirmed improved response (MR4.5, 54% and 52% in two nilotinib arms compared to 31% with imatinib). Long‐term toxicities were not significantly different, making this a valuable option [3, 4, 8]. Our patient achieved and maintained complete molecular response (MR4.5). Enest freedom study evaluated the potential for treatment‐free remission in patients with sustained complete molecular response (MR‐4.5). After 2 years of observation without treatment, 51.6% (confidence interval 44.2%‐58.9%) remained in major molecular response or better (BCR‐ABL ≤ 0.1%) [17].

Our patient tolerated nilotinib therapy without significant toxicities. Asymptomatic and transient elevations of pancreatic enzymes were noted that resolved without dose reduction or discontinuation of nilotinib. Asymptomatic elevations in pancreatic enzymes have been observed in around a third of patients including grade 3‐4 in 18% of patients, however this elevation is not usually associated with acute pancreatitis. Median time from initiation of nilotinib to elevation of pancreatic enzymes was 3 months. Pancreatic enzyme elevation was isolated or transient in most patients. Occasional drug interruption was necessary [10]. The underlying reason is unclear; suggested mechanisms include inhibition of non‐receptor tyrosine kinase C‐abl, calcium release from intracellular stores, and accumulation of fatty acids in the acinar cells [11].

While asymptomatic pancreatic elevations are relatively common after initiation of nilotinib, acute pancreatitis is a rare side effect (around 1%), and occurs relatively early within weeks or months. Acute pancreatitis has been observed after initiation of other tyrosine kinase inhibitors in the therapy of CML and other malignancies. Delayed occurrence of pancreatitis has not been reported thus far [10, 11, 12, 13].

In our patient, acute abdominal symptoms, magnitude of pancreatic enzyme elevation, and CT imaging were consistent with diagnosis of acute pancreatitis based upon the Atlanta criteria and graded as mild based on the revised Atlanta criteria [14]. She was diagnosed with acute drug‐induced pancreatitis after exclusion of other causes. Drugs account for 2% of acute pancreatitis cases, which is usually mild to moderate and resolves with discontinuation of drugs.

Drugs other than nilotinib strongly implicated in acute pancreatitis on the patient's medication list included lisinopril and furosemide [15]. Nilotinib was discontinued on day 2 of hospitalization and the patient showed prompt reduction in enzyme elevation and clinical symptoms. Lisinopril and furosemide therapy were continued, despite which the patient has not had any recurrence of pancreatitis.

**FIGURE 2:**
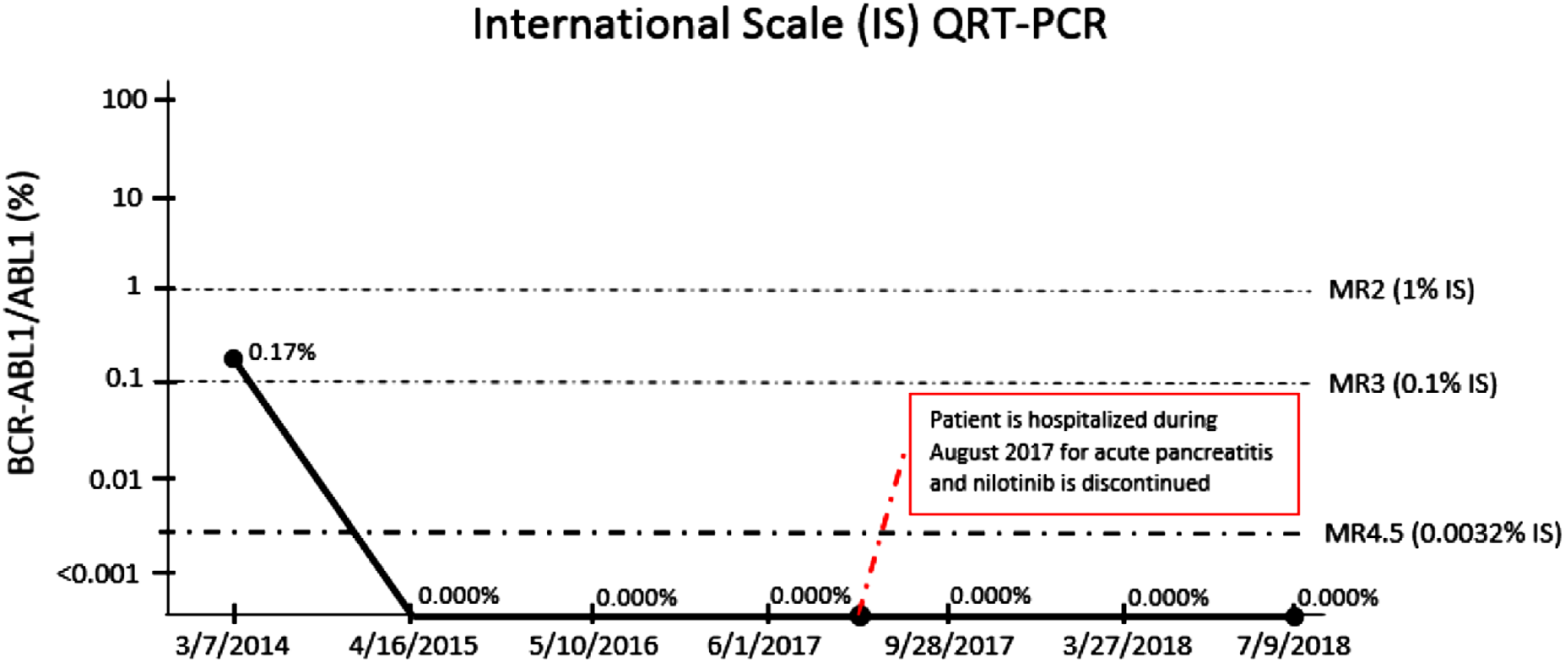
patient's timeline for QRT‐PCR showing treatment response and monitoring after discontinuing nilotinib

In our patient, nilotinib was discontinued and no alternate tyrosine kinase inhibitor was initiated because the recurrence of pancreatitis on the initiation of alternate agents has not been well studied. Acute pancreatitis appears to be a class effect of all tyrosine kinase inhibitors. Discontinuation of nilotinib has been studied in one phase 2 trial, Enest freedom study, involving patients with sustained deep molecular response (MR4.5). Molecular response was sustained for more than 12 months in more than 50% of the patients. In addition, the majority of patients (>90%) re‐initiated on nilotinib due to loss of major molecular response (MR3.0) regained it [16, 17]. On close monitoring, our patient continues to show complete molecular response more than 2 years after discontinuation of nilotinib (Figure 2).
